# Intensive glycemic control and kidney disease risk: insights on hierarchical composite endpoint from a randomized clinical trial

**DOI:** 10.3389/fmed.2025.1636392

**Published:** 2025-09-03

**Authors:** Zhaojie Song, Haibao Xu, Jiaheng Zhang, Yezhou Liu, Chao Li, Tao Chen, Sujuan Guo, Ni Zhu

**Affiliations:** ^1^School of Public Health, Xi’an Jiaotong University Health Science Center, Xi’an, China; ^2^School of Economics and Finance, Xi’an Jiaotong University, Xi’an, China; ^3^Shaanxi International Trust Limited-Liability Company, Xi’an, China; ^4^Key Laboratory of Environment and Genes Related to Diseases, Xi'an Jiaotong University, Ministry of Education, Xi'an, Shaanxi, China; ^5^Department of Clinical Sciences, Liverpool School of Tropical Medicine, Liverpool, United Kingdom; ^6^Infectious Disease Department, Baoji Central Hospital, Baoji, China; ^7^Shaanxi Provincial Center for Disease Control and Prevention, Xi’an, China

**Keywords:** type 2 diabetes mellitus, intensive glycemic control, diabetic kidney disease, hierarchical composite endpoint, cardiovascular risk, cardio-renal syndrome, Win Odds

## Abstract

**Background:**

Clinical trials of intensive glycemic control in patients with type 2 diabetes mellitus (T2DM) and high cardiovascular risk have reported inconsistent findings regarding chronic kidney disease (CKD) outcomes, partly due to heterogeneity in event definitions and reliance on conventional time-to-first-event analysis. This study aimed to evaluate the renal effects of intensive glycemic control using a hierarchical composite endpoint (HCE) ranked by clinical severity and analyzed via the Win Odds (WO) method.

**Method:**

This post-hoc analysis included patients from the Action to Control Cardiovascular Risk in Diabetes (ACCORD) glycemia trial. We employed the win ratio statistical method to estimate the treatment effects on HCE, defined as a ranked composite of all-cause mortality, kidney failure, sustained estimated glomerular filtration rate (eGFR) declines of 57, 50, and 40% from baseline, persistent eGFR < 15 mL/min/1.73 m^2^, and eGFR slope. The effects of intensive glycemic control on individual HCE components and various composite kidney endpoints was assessed by Cox regression models.

**Results:**

Among the 9,848 participants, sustained 40% eGFR decline was the most frequent renal event in the hierarchical composite. Intensive glucose control was not associated with a significant difference in the HCE compared to standard therapy (WO = 1.03, 95% CI: 0.99–1.07). This finding was consistent with results from Cox regression (HR = 1.05, 95% CI: 0.97–1.13) and across individual components of the composite endpoint.

**Conclusion:**

In individuals with T2DM at high risk for cardiovascular disease, intensive glycemic control does not demonstrate a significantly detrimental effect on hierarchical composite kidney outcomes.

## 1 Introduction

T2DM is a chronic condition associated with a range of serious complications, including kidney damage, and peripheral neuropathy (1–4). Effective management of blood glucose levels is crucial for mitigating these complications (5, 6). Although intensive glycemic control appears to confer a near-lifelong benefit of cardiovascular health (7), the growing use of novel pharmacological agents and combination therapies has raised concerns regarding potential adverse effects on renal function (8, 9).

The ACCORD trial focusing on the assessment of conventional kidney-related outcomes have found that intensive glucose-lowering therapy are effective in reducing the risk of early-stage renal dysfunction (microalbuminuria) (10). However, these interventions have not been shown to significantly impact the progression to advanced stage of kidney diseases (kidney failure and all-cause mortality) (11, 12). In addition, previous studies analyzing the impact of intensive glucose-lowering interventions on composite renal endpoints have predominantly focused on the first occurrence of any endpoint, neglecting the varying severity levels of different outcomes. Consequently, there remains a lack of robust evidence regarding the effects on renal system function.

To address the limitations of using conventional endpoints or composite endpoints without considering severity gradations, this study employs the WOs measure within the win ratio method, incorporating multiple indicators that reflect the progression of renal dysfunction from early stages to mortality, providing a more comprehensive assessment of the impact of intensive glycemic control throughout the progression of kidney disease.

## 2 Methods

### 2.1 Trial design and oversight

This study is a *post hoc* analysis of ACCORD BioLINCC dataset obtain from the NIH upon approval. The design and conduct of the randomized controlled ACCORD trial have been reported previously (13). Briefly, the ACCORD trial was a rigorously designed double two-by-two factorial study. Middle-aged individuals (mean age 62.2 years) diagnosed with diabetes at high cardiovascular risk were assigned to either intensive therapy that targeted HbA1c lower than 6% (42 mmol/mol) or standard group that targeted HbA1c 7–7.9% (53–63 mmol/mol). The ACCORD glycemia trial was halted prematurely after a mean duration of 3.7 years, due to the Data Safety Monitoring Board’s observation of higher mortality rates in the intensive glucose-lowering group (14). This analysis was approved by the institutional review board (IRB) of the participating institution, and the Ethical Review Board of the First Affiliated Hospital of Xi’an Jiaotong University waived the need for additional ethical approval (MC-KYLLSL-2023-005).

### 2.2 eGFR measurement and endpoint definitions

eGFR was calculated in a standardized manner using the Chronic Kidney Disease Epidemiology Collaboration creatinine equation (15). Participants from the ACCORD glycemia trial with baseline eGFR measurements and two or more follow-up eGFR data were included in analyses. We defined the HCE to capture the clinical severity of kidney outcomes, which encompass all-cause mortality, end-stage renal disease (ESRD) requiring renal replacement therapy or transplantation, a sustained eGFR of less than 15 mL/min/1.73 m^2^ for at least 30 days, sustained declines in eGFR of 57, 50%, or 40% (each confirmed by a subsequent measurement ≥30 days later), and the eGFR slope (16, 17). In accordance with KDIGO recommendations (18), sustained eGFR <15 mL/min/1.73 m^2^ was incorporated into the ESRD category in the hierarchical composite for Win Odds analysis. The analysis will be restricted to events and eGFR measurements up to a specified cut-off of 3 years following randomization. The acute event will be considered to have occurred at the initial visit, which is Day 14 (this will be divided by 360 to convert to years). The coefficient for the chronic phase will also be derived, which is the proportion of the length of the chronic phase (total follow-up minus the acute phase) divided by the total follow-up for an individual (19). This will be used to derive the total GFR slope from the two-slope power-of-the-mean model.

### 2.3 Statistical analysis

Baseline characteristics of participants were presented as frequencies with percentages, means with standard deviation, or medians with interquartile ranges. The HCE was analyzed using WOs, an adaptation of win ratio (20) to include ties (a tie is considered a half loss and a half win for each group). For each patient pair, the winner was identified sequentially based on the severity of clinical events, from the most severe to the least severe. If one patient experienced an event, that patient was deemed the winner, with earlier occurrence further conferring an advantage; if neither patient experienced an event, the pair was considered a tie (21). The hierarchical comparison of HCE components is provided in Supplementary Table 1.

WOs were computed by summing the wins and half of the ties, then dividing by the total losses plus half of the ties (22). Maraca plot was used to visualize the contribution of components of HCE over time, combining time-to-event outcomes with a continuous outcome (23). In the Maraca plot, the x-axis represents a consistent follow-up duration for each dichotomous outcome, arranged by severity. The continuous outcome covers the entire range of possible values. The width of each component corresponds to its proportional contribution to the composite outcome. The Win Odds framework accounts for clinical severity by applying a predefined hierarchical structure to composite outcomes. As part of the sensitivity analyses, the hierarchy was extended to include albuminuria components defined by the urinary albumin-to-creatinine ratio (uACR), including incident macroalbuminuria (uACR ≥300 mg/g) and microalbuminuria (uACR 30–299 mg/g), which were placed after sustained eGFR decline events. Cox proportional hazards models were also used to evaluate the effect of intensive versus standard intervention on the time to first event across binary components of HCE, with results presented as HRs and 95% CIs. A two-slope mixed-effects model was applied to evaluate treatment effects on overall eGFR slope, with results presented as means and 95% CIs. The Cumulative incidence functions and Kaplan–Meier (KM) curves were used to estimate the probability of achieving each HCE component. All analyses were performed using Stata version 18.0 and R version 4.4.2.

## 3 Results

After excluding participants without baseline eGFR data (n = 48) or those with fewer than two eGFR measurements (n = 355), the analysis included a total of 9,848 participants, comprising 4,931 in the standard treatment group and 4,917 in the intensive treatment group (Supplementary Figure 1).

The median age was 62.7 years, and 38.3% participants were female. In terms of disease history, 54.6% used aspirin, and 34.9% had a history of cardiovascular disease. The prevalence of dyslipidemia and hypertension was 94.2 and 96.4%, respectively. Baseline laboratory measurements showed a mean eGFR of 91.1 mL/min/1.73 m^2^, with 92.0% having an eGFR more than 60 mL/min/1.73 m^2^. Blood pressure, LDL, HDL, BMI, and glucose values were comparable across groups (Table 1).

**Table 1:** Baseline characteristics by randomized group.

Variable	Standard Group	Intensive Group	Total
	(*N* = 4,931)	(*N* = 4,917)	(*N* = 9,848)
Demography			
Age	62.75 (6.62)	62.73 (6.61)	62.74 (6.61)
Gender			
Male	3,044 (61.71)	3,031 (61.64)	6,075 (61.68)
Female	1,889 (38.29)	1,886 (38.36)	3,775 (38.32)
Race			
White	3,098 (62.8)	3,080 (62.64)	6,178 (62.72)
Black	911 (18.47)	941 (19.14)	1,852 (18.8)
Hispanic	358 (7.26)	341 (6.94)	699 (7.1)
Other	566 (11.47)	555 (11.29)	1,121 (11.38)
Education			
High School	1,318 (26.73)	1,292 (26.29)	2,610 (26.51)
College	1,628 (33.02)	1,609 (32.74)	3,237 (32.88)
Bachelor	1,315 (26.67)	1,255 (25.54)	2,570 (26.11)
Disease history			
Aspirin	2,682 (54.40)	2,696 (54.83)	5,378 (54.61)
Smoking	575 (11.66)	610 (12.41)	1,185 (12.03)
Drinking	1,202 (24.38)	1,167 (23.74)	2,369 (24.06)
Cardiovascular	1,694 (34.35)	1,739 (35.37)	3,433 (34.86)
Dyslipidemia	4,645 (94.16)	4,639 (94.35)	9,284 (94.25)
Hypertension	4,740 (96.09)	4,752 (96.64)	9,492 (96.37)
Laboratory			
eGFR, mL/min per 1.73 m^2^	91.36 (28.51)	90.8 (25.75)	91.08 (27.17)
eGFR (median, IQR)	89.7 (76–105.1)	89.5 (74.8–104.5)	89.6 (75.4–104.8)
eGFR, mL/min per 1.73 m^2^			
≥ 60	4,554 (92.32)	4,510 (91.72)	9,064 (92.02)
<60	377 (7.64)	407 (8.28)	784 (7.96)
SBP (mmHg.)	136.43 (17.19)	136.17 (16.87)	136.3 (17.03)
DBP (mmHg)	74.95 (10.68)	74.77 (10.57)	74.86 (10.62)
LDL C (mg/dL)	104.91 (33.78)	104.74 (33.82)	104.82 (33.8)
HDL C (mg/dL)	41.88 (11.39)	41.79 (11.63)	41.83 (11.51)
BMI (kg/m^2^)	32.24 (5.38)	32.24 (5.41)	32.24 (5.39)
Glucose (mg/dL.)	175.8 (56.34)	174.65 (55.59)	175.22 (55.97)
Potassium (mmol/L)	4.47 (0.5)	4.48 (0.44)	4.47 (0.47)

Values are *N* (%) or mean (SD).

**Figure 1:**
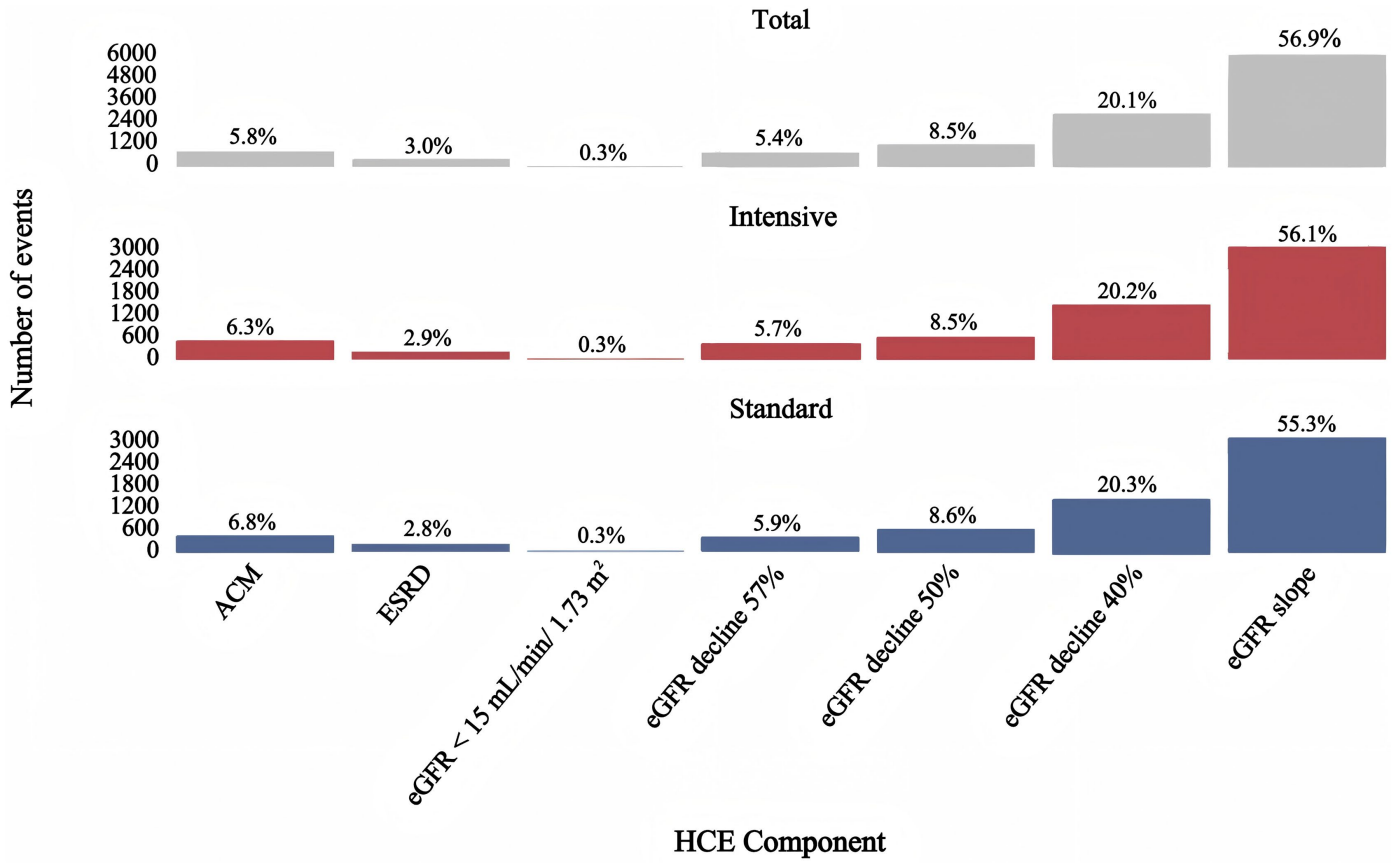
Number (%) of individual components of the renal composite endpoint in the ACCORD study in all persons and in the intensive glycemic intervention and standard glycemic intervention groups. ACM, all-cause mortality; ESRD, end stage renal disease.

A decrease in eGFR slope is the main components in the HCE. Among participants in the intensive treatment group, the most frequently observed outcome was a decline in eGFR slope (56.1%), followed by a 40 and 50% reduction in eGFR. A similar trend was observed in the standard treatment group, where 55.3% of participants experienced a decline in eGFR slope. Severe eGFR declines to below 15 mL/min/1.73 m^2^ were rare, occurring in only 0.3% of participants in both groups (Figure 1).

**Figure 2:**
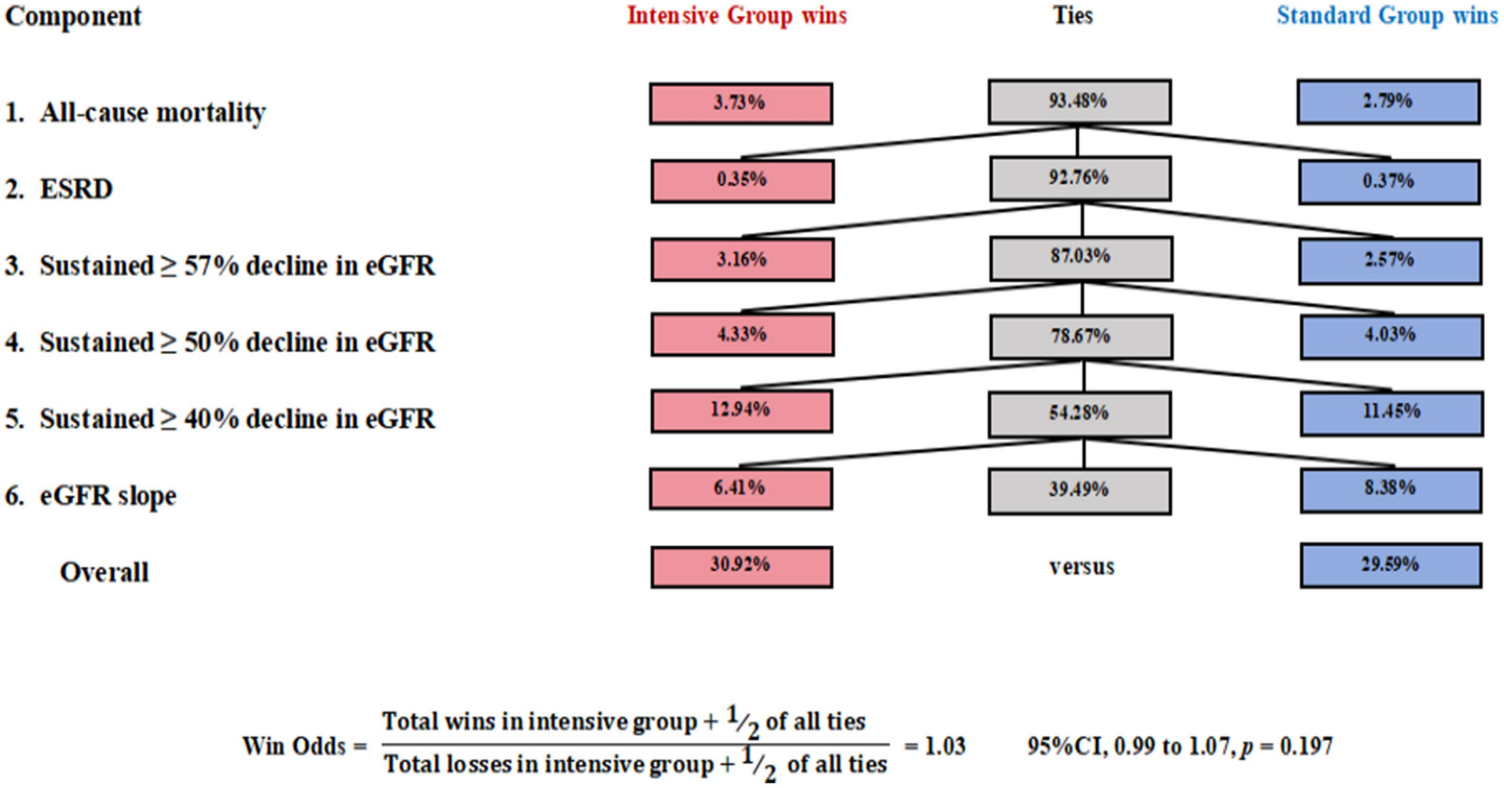
The Win Odds in the ACCORD trial. Win Odds were computed in a hierarchy: all-cause mortality; ESRD (including sustained eGFR <15 mL/min/1.73 m^2^); ≥57%, ≥50%, and ≥40% decline in eGFR; and eGFR slope. eGFR slope decline.

In this study, all patients who experienced persistent eGFR <15 mL/min/1.73 m^2^ ultimately progressed to ESRD. Therefore, this event was incorporated into the higher-priority ESRD tier and did not serve as an independent level of comparison in the Win Odds analysis. As shown in Figure 2, tie rates exceeded 54.28% for all components except eGFR slope, which had a lower tie percentage of 39.49%, indicating greater discriminatory power. Using a tie-adjusted formula, the overall Win Odds was 1.03 (95% CI, 0.99 to 1.07), suggesting a slightly favorable trend for the placebo group compared to the intensive treatment group in terms of the HCE.

The Maraca plot demonstrates more dichotomous outcomes in the active group compared to placebo group, indicating that while the median rate of eGFR decline (shift to the right in the maraca plot) in active compared with the placebo group, the overall difference between groups was not statistically significant (Supplementary Figure 2).

Among 9,848 patients with type 2 diabetes at high cardiovascular risk receiving glucose-lowering therapy in the ACCORD trial, there were 1,987 cases of a 40% decline in eGFR, 840 cases of a 50% decline, 557 cases of a 57% decline, 33 cases of persistent eGFR < 15 mL/min/1.73 m^2^, 286 cases requiring dialysis, and 622 died. For the composite kidney outcome, which included all-cause mortality, ESRD, and a 40% eGFR decline, the HR was 1.05 (95%CI, 0.97 to 1.13). Similarly, for the composite endpoint including all-cause mortality, ESRD, and a 57% eGFR decline, the HR was 1.07 (95%CI, 0.97 to 1.18). When analyzing the composite outcome of all-cause mortality, ESRD, and a 50% decline in eGFR, the HR was 1.09 (95%CI, 0.98 to 1.22). The WO for the composite kidney outcome was 1.03 (95%CI, 0.99 to 1.07), again revealing no significant difference between the treatment groups (Table 2). Across multiple HCE definitions, almost no significant differences were observed between treatment groups. For the relatively comprehensive HCE (Tiers 1–8), the Win Odds was 1.00 (95% CI, 0.95 to 1.04), and the HR was 1.02 (95% CI, 0.95 to 1.08). When albuminuria-related components (Tiers 6 and 7) were included, treatment effects remained neutral. A slight benefit was observed for the combination of Tiers 7 and 8, with a Win Odds of 1.07 (95% CI, 1.02 to 1.12) (Supplementary Figure 4). Kaplan–Meier survival curves (Supplementary Figure 3) showed minimal divergence between the treatment groups for kidney-related events, supporting the conclusion that intensive glycemic control did not significantly affect kidney outcomes.

**Table 2:** Comparison of time to first event analysis and Win Odds.

Treatment comparisons	Intensive vs. standard	
	*n*	HR (95% CI)
Event		
Tier 1: All-cause mortality	622	1.18 (1.01 to 1.38)
Tier 2: ESRD	288	0.94 (0.74 to 1.18)
Tier 3: eGFR <15 mL/min per 1.73 m^2^	33	0.95 (0.48 to 1.88)
Tier 4: 57% eGFR decline	557	1.11 (0.94 to 1.31)
Tier 5: 50% eGFR decline	840	1.02 (0.89 to 1.17)
Tier 6: 40% eGFR decline	1,987	1.02 (0.93 to 1.11)
Tier 7: eGFR slope^a^	−0.90 (−2.16 to 0.36)	
Treatment effect composite end point		
HR (Tier 1 or 2 or 3 or 6)	1.05 (0.97 to 1.13)	
HR (Tier 1 or 2 or 3 or 5)	1.07 (0.97 to 1.18)	
HR (Tier 1 or 2 or 3 or 4)	1.09 (0.98 to 1.22)	
HR (Tier 1 to 6)	1.04 (0.97 to 1.12)	
WOs^b^	1.03 (0.99 to 1.07)	

^a^Estimated means (95% CIs) derived from the mixed-effects model. ^b^Win Odds were computed in a hierarchy: all-cause mortality; ESRD; sustained eGFR <15 mL/min/1.73 m^2^; ≥57%, ≥50%, and ≥40% decline in eGFR; and eGFR slope.

## 4 Discussion

This study used the win ratio statistical method to compare the effects of intensive and standard glucose-lowering treatments on HCE, finding no significant difference. Further analyses using Cox modeling across various kidney outcome combinations of differing severity also showed no significant effects.

Some studies investigating intensive glycemic control in patients with T2DM have showed its effectiveness in reducing the risk of early kidney damage. Specifically, the ACCORD trial, ADVANCE trial and the EDIC study reported a lower incidence of microalbuminuria in the intensive treatment group compared to the conventional group (24–26). Likewise, R. Bilous’ analysis of the UKPDS found that tighter glycemic control reduced the relative risk of proteinuria and significantly lowered the proportion of patients with a twofold increase in plasma creatinine levels (27). Furthermore, in the VADT-F trial, a significantly higher proportion of participants in the intensive treatment group retained normal kidney function at study completion (28). Considering the above, the use of isolated and non-continuous renal endpoint endpoints without a clear severity ranking may cause heterogeneities (29). By employing HCE analysis in our study, we conducted a systematic assessment by integrating and hierarchically ranking multiple renal outcomes, including all-cause mortality, ESRD, eGFR decline, and eGFR slope, based on their clinical severity, revealing that intensive glycemic control does not exert significant adverse effects on renal function.

Moreover, while several studies have utilized composite renal outcomes, they predominantly relied on first-event analyses, which may overlook clinically more severe but later-occurring events, such as ESRD or mortality. For instance, a retrospective cohort study found that intensive glucose lowering did not reduce the risk of persistent eGFR below 15 mL/min/1.73 m^2^, doubling of serum creatinine levels, or ESRD (30). Similarly, a *post hoc* analysis of the ACCORD trial showed no significant reduction in the need for dialysis, or death from any cause in patients who received aggressive treatment (10). To address this limitation, our study utilized the WOs metric within the win ratio methodology, which prioritizes events based on clinical significance rather than chronological occurrence. This approach mitigates biases associated with traditional first-event analyses and is consistent with KDIGO and ERBP guidelines for standardized renal endpoint monitoring (18, 31, 32).

Our findings demonstrate the complementary strengths of Cox regression and the Win Odds approach in evaluating renal composite outcomes. While Cox models consider the time to first event irrespective of clinical severity, Win Odds emphasizes early and clinically significant events within a fixed 3-year window. This difference explains the limited contribution of rare but severe events, such as sustained eGFR <15 mL/min/1.73 m^2^, in the Win Odds analysis. Notably, when albuminuria components were incorporated into the hierarchy, Win Odds revealed modestly favorable trends, highlighting the sensitivity of uACR as an early indicator of kidney injury. This is consistent with previous findings, such as those by An et al. (33), which reported that intensive HbA1c reduction may coincide with short-term eGFR decline in patients with elevated uACR. Given the limited number of events for certain uACR-related tiers, this trend should be interpreted cautiously due to the potential for false-positive findings. Collectively, these results underscore the value of hierarchical composite frameworks for capturing nuanced treatment effects across heterogeneous renal outcomes and the importance of including sensitive markers like uACR in future endpoint definitions.

Although the use of a composite outcome strengthens the validity of our findings, the relatively homogeneous patient population may limit the generalizability of the results. Additionally, the relatively short follow-up period restricts the ability to comprehensively evaluate the long-term effects of intensive glycemic control on renal function. Finally, the inclusion of renal status may be incomplete in our study, underscoring the need for more comprehensive assessments in future research.

## 5 Conclusion

Using hierarchical definitions for kidney endpoints, this study found no significant difference between the intensive and standard glucose-lowering groups. Sensitivity analyses consistently supported this conclusion, suggesting that intensive glycemic control may not provide a clear advantage over standard treatment in improving renal outcomes.

## Data Availability

The individual-level, de-identified participant data from the Action to Control Cardiovascular Risk in Diabetes (ACCORD) trial are publicly available through the Biologic Specimen and Data Repository Information Coordinating Center (BioLINCC). The dataset can be accessed at: https://biolincc.nhlbi.nih.gov/studies/accord/Repository: BioLINCC (NHLBI) Accession Number: 2003-001.
