# Supplementary material for: Intensive glycemic control and kidney disease risk: insights on hierarchical composite endpoint from a randomized clinical trial
Source: Front Med (Lausanne). 2025 Sep 3;12:1636392. doi: 10.3389/fmed.2025.1636392 (PMC12440872; doi:10.3389/fmed.2025.1636392)
Supplement: Supplementary file 1 [file Data_Sheet_1.docx]

Supplementary Material

# Supplementary Figures and Tables

## Supplementary Figures


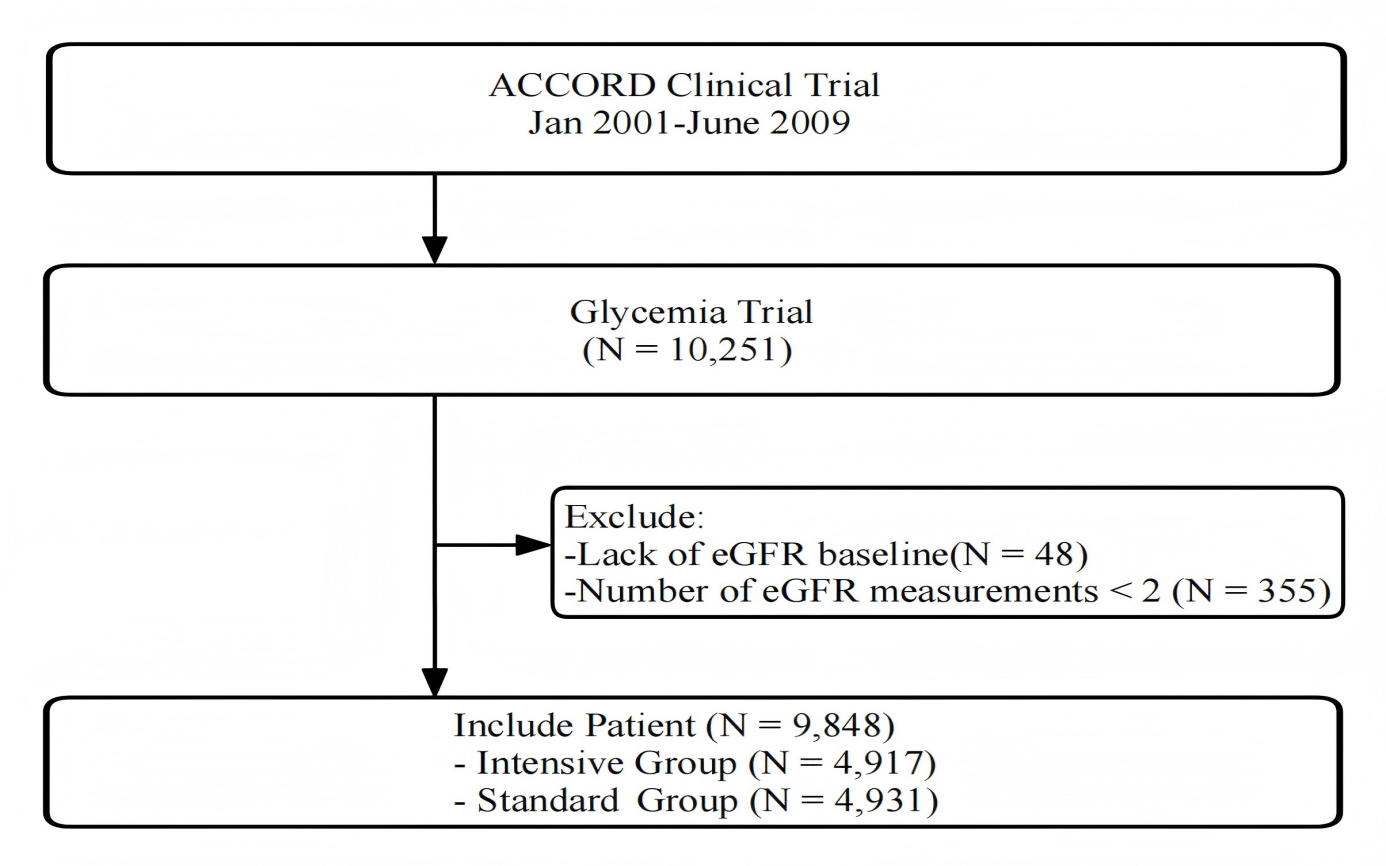


**Supplementary Figure 1. Flowchart of Study Selection.**

**
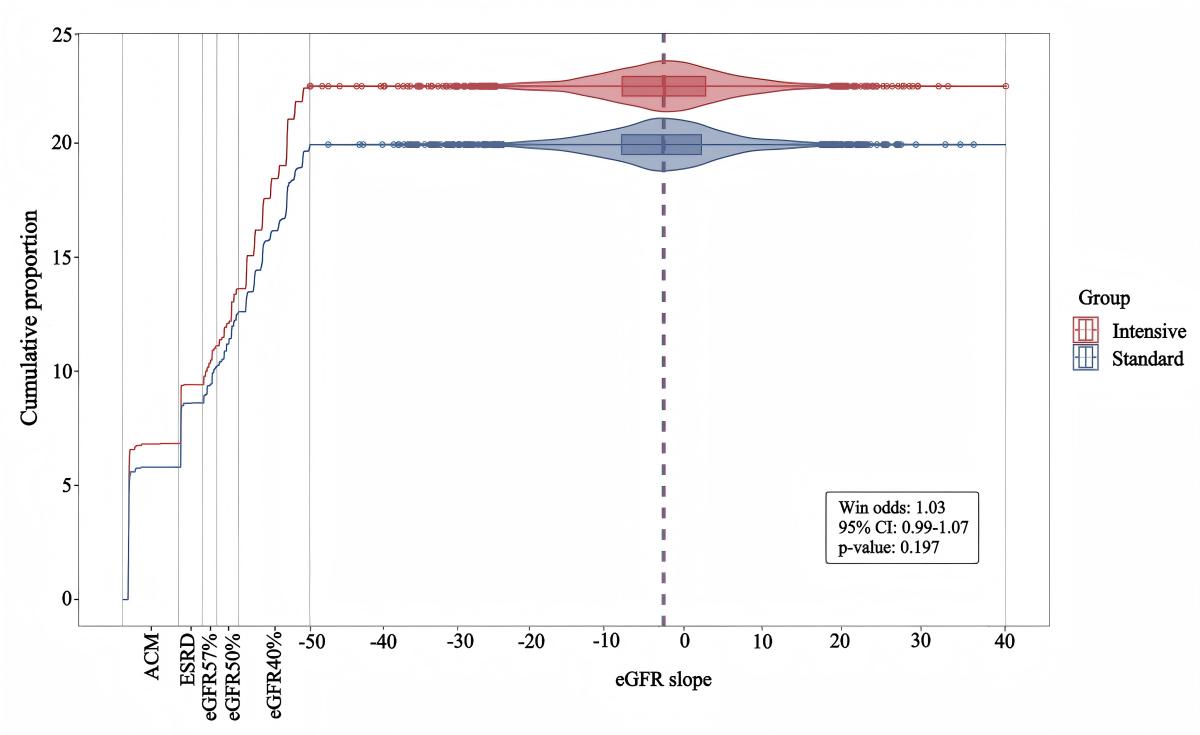
**

**Supplementary Figure 2. The maraca plot of kidney HCE.**

ACM, all-cause mortality; ESRD, end stage renal disease; eGFR57%, Sustained ≥ 57% decline in eGFR; eGFR50%, Sustained ≥ 50% decline in eGFR; eGFR40%, Sustained ≥ 40% decline in eGFR.


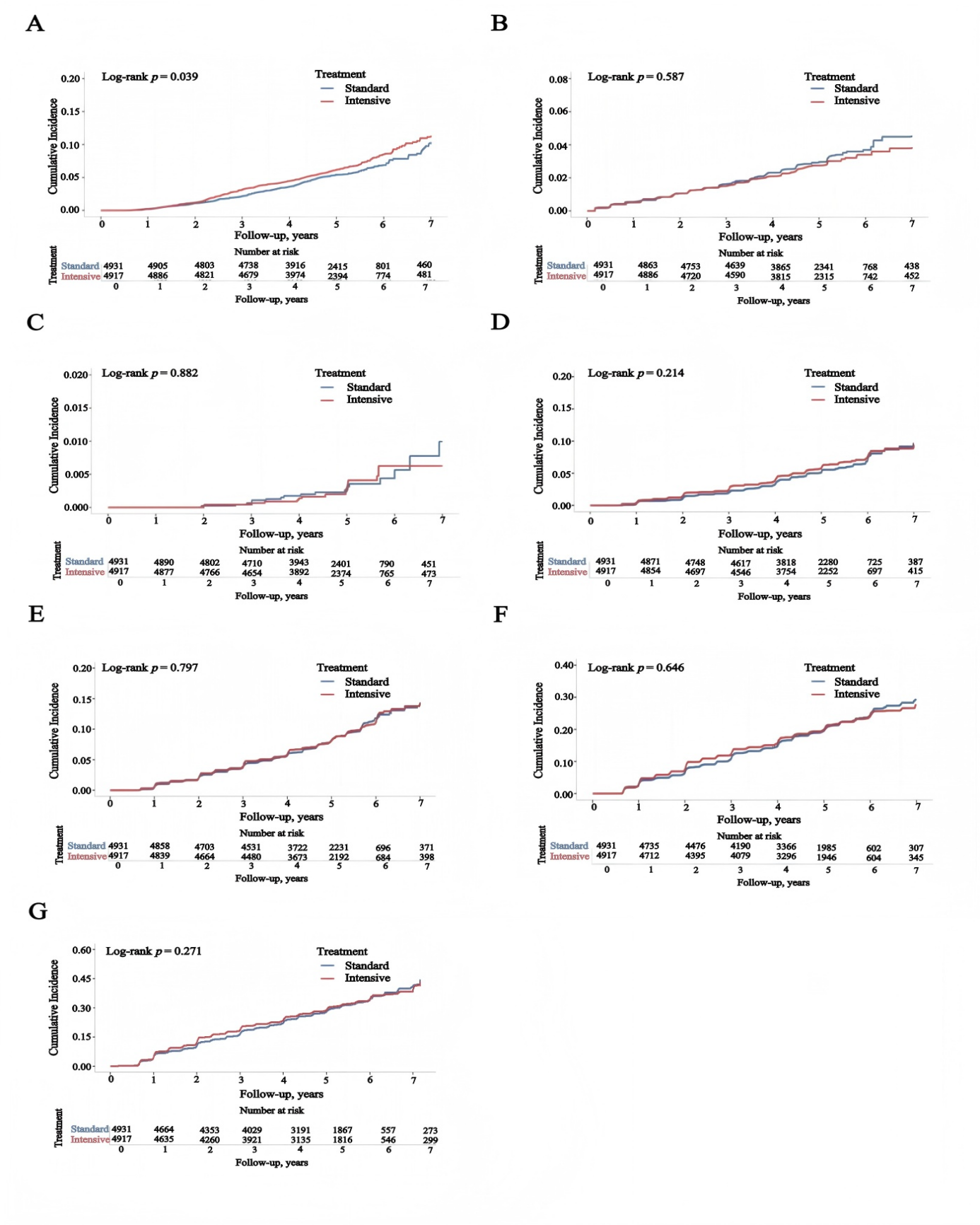


**Supplementary Figure 3. Kaplan-Meier Curve of the hierarchical composite kidney endpoint in the ACCORD trial.**

(A) All-Cause Mortality. (B) End stage renal disease. (C) Sustained eGFR< 15 mL/min/1.73 m^2^. (D) Sustained ≥ 57% decline in eGFR. (E) Sustained ≥ 50% decline in eGFR.. (F) Sustained ≥ 40% decline in eGFR. (G) Composite CKD Endpoint.


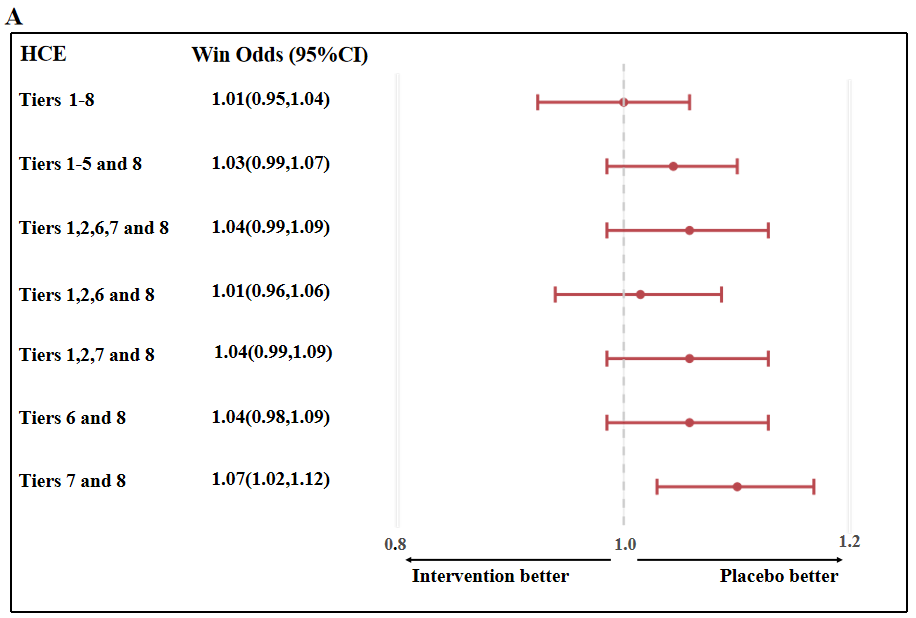

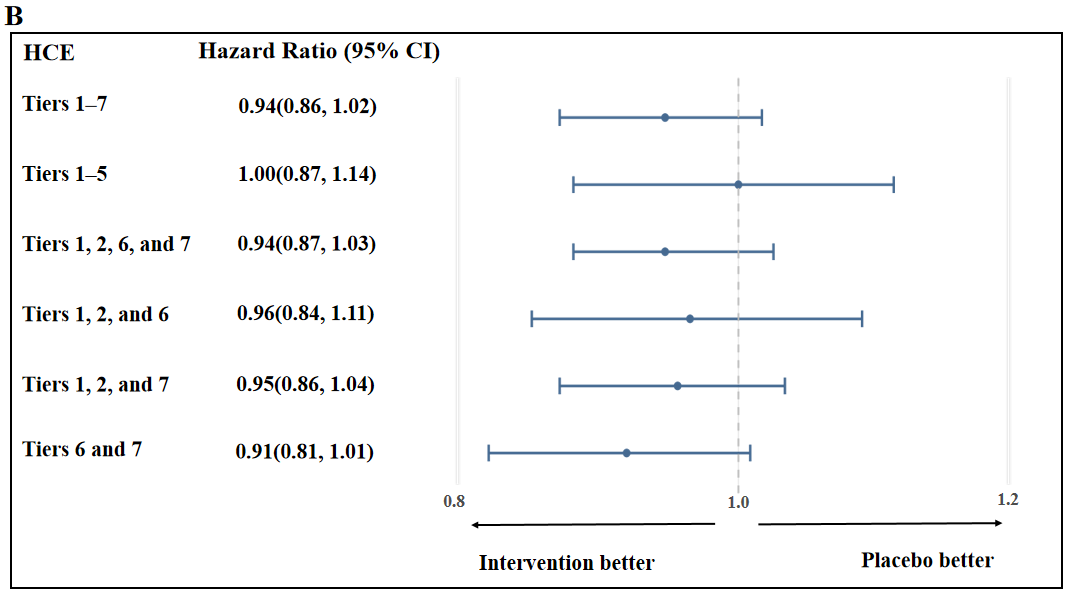


**Supplementary Figure 4. Comparison of treatment effects using Win Odds and Cox regression across hierarchical composite kidney outcomes.**

(A) Win Odds and 95% confidence intervals (CIs) for different HCE definitions, incorporating various combinations of kidney-related events from Tiers 1 to 8.

(B) Hazard Ratios and 95% CIs from Cox regression models for the same composite endpoints. Win Odds accounts for severity ranking; Cox regression models time-to-event.

## Supplementary Table

**Supplementary Table 1. Principle of Hierarchical Comparison of Events for a Pair of Patients, One in the Intensive Arm and One in the Standard Arm**

|  | **Outcome** |
| --- | --- |
| **First comparison:** |  |
| Patient in Intensive arm dies first | Intensive wins |
| Patient in Standard arm dies first | Standard wins |
| Both patients die on the same day | Tie |
| Neither of the patients die | Go to second comparison |
| **Second comparison:** |  |
| Patient in Intensive arm has ESRD^*^ first | Intensive wins |
| Patient in Standard arm has ESRD first | Standard wins |
| Both patients have ESRD on the same day | Tie |
| Neither of the patients have ESRD | Go to third comparison |
| **Third comparison:** |  |
| Patient in Intensive arm has eGFR < 15 ^a^ first | Intensive wins |
| Patient in Standard arm has eGFR < 15 first | Standard wins |
| Both patients have a eGFR < 15 on the same day | Tie |
| Neither of the patients have a eGFR < 15 | Go to fourth comparison |
| **Fourth comparison:** |  |
| Patient in Intensive arm has 57% eGFR decline first | Intensive wins |
| Patient in Standard arm has 57% eGFR decline first | Standard wins |
| Both patients have a 57% eGFR decline on the same day | Tie |
| Neither of the patients have a 57% eGFR decline | Go to fifth comparison |
| **Fifth comparison:** |  |
| Patient in Intensive arm has 50% eGFR decline first | Intensive wins |
| Patient in Standard arm has 50% eGFR decline first | Standard wins |
| Both patients have a 50% eGFR decline on the same day | Tie |
| Neither of the patients have a 50% eGFR decline | Go to sixth comparison |
| **Sixth comparison:** |  |
| Patient in Intensive arm has 40% eGFR decline first | Intensive wins |
| Patient in Standard arm has 40% eGFR decline first | Standard wins |
| Both patients have a 40% eGFR decline on the same day | Tie |
| Neither of the patients have a 40% eGFR decline | Go to last comparison |
| **Seventh comparison:** |  |
| Patient in Intensive arm has faster progressing eGFR slope | Intensive wins |
| Patient in Standard arm has faster progressing eGFR slope | Standard wins |
| Patients have similar eGFR slope | Tie |

* End stage renal disease.

^a^ eGFR < 15 mL/min/1.73 m^2^.
